# The impact of restrictive blood transfusion on the safety of patients with esophageal varices after endoscopic variceal ligation: A single-center retrospective study

**DOI:** 10.1097/MD.0000000000039407

**Published:** 2024-08-16

**Authors:** Yufeng Chen, Wen Ming, Jingjing Chen, Xi Wang, Guobin He

**Affiliations:** a Department of Gastroenterology, Affiliated Hospital of North Sichuan Medical College, Nanchong, Sichuan, China.

**Keywords:** endoscopic variceal ligation, hemoglobin, liver cirrhosis, rebleeding, restrictive blood transfusion

## Abstract

An investigation was conducted to examine the impact of restrictive blood transfusion on the safety of early rebleeding following endoscopic variceal ligation (EVL) in patients with liver cirrhosis. Data were collected from patients with cirrhosis and esophageal varices who underwent EVL at the Affiliated Hospital of North Sichuan Medical College between September 2021 and March 2023. Clinical information, including serum albumin levels, hemoglobin (Hb) levels, liver function classification, and the occurrence of early rebleeding, was recorded. Patients were divided into 2 groups based on their Hb levels: 60 g/L to 90 g/L (restrictive blood transfusion) or Hb ≥ 90 g/L after EVL. The impact of restrictive transfusion on the post-ligation safety of EVL was observed. A total of 246 cirrhotic patients were included in the analysis. Significant differences were found in Hb levels, liver function classification, early rebleeding rates, length of hospital stay, and hospitalization expenses between the restrictive transfusion and Hb ≥ 90 g/L groups. The early rebleeding rate was significantly varied between the groups with different Hb levels after EVL. Multivariate logistic analysis revealed that restrictive blood transfusion (OR = 4.61, 95% CI: 1.06–19.99; *P* = .041), Hb (OR = 0.96, 95% CI: 0.95–0.97; *P* < .001), and Child-Pugh class C (OR = 6.37, 95% CI: 1.28–31.67; *P* = .024) were identified as independent factors influencing early rebleeding. Our findings suggest that the risk of early rebleeding in cirrhotic patients after EVL may be increased by restrictive blood transfusion, and this should be further investigated in future research.

## 1. Introduction

Liver cirrhosis commonly accompanies varying degrees of portal hypertension, with esophageal varices being frequent endoscopic manifestations of this condition. Late rupture risk is notably high in these varices. Esophageal variceal bleeding (EVB) accounts for approximately 70% of all upper gastrointestinal bleeding cases in patients with portal hypertension.^[1,2]^ The annual incidence of EVB ranges from 10% to 15%, and the 6-week mortality rate can reach 20%. EVB is characterized by its acute onset, rapid progression, and high mortality, defining it as the principal decompensation event in cirrhosis.^[3–5]^ While both endoscopic variceal ligation (EVL) and sclerotherapy are effective in treating EVB, EVL offers advantages such as reduced need for repeated endoscopic interventions and lower complication rates.^[6–8]^ International guidelines, such as the Baveno-VII consensus, primarily recommend EVL as the initial endoscopic therapy for acute variceal bleeding and as the primary or secondary prevention option when nonselective beta blockers are contraindicated or not tolerated.^[6,9–11]^

For patients experiencing acute EVB, blood transfusion is another crucial aspect of managing these patients. Red blood cell (RBC) transfusions are indicated when hemoglobin (Hb) levels drop to ≤70 g/L, and hemodynamics are stable, and there is no history of cardiovascular disease.^[6,10,12]^ However, for individuals experiencing upper gastrointestinal bleeding, particularly those with EVB, excessive or rapid blood transfusion or infusion can potentially raise portal pressure and lead to rebleeding, particularly in cases of preexisting portal hypertension. Many prior studies^[13–15]^ found that the restrictive transfusion strategy is as safe and effective as the liberal transfusion approach, and a restrictive RBC transfusion strategy is advised. The Baveno-VII consensus recommends maintaining Hb levels between 70 g/L and 80 g/L.^[9]^ The European Society of Gastrointestinal Endoscopy Guidelines from 2015 propose a target Hb range of 70 g/L to 90 g/L,^[10]^ while the Chinese guideline recommends maintaining it between 60 g/L and 70 g/L.^[6]^

While most existing studies on restrictive transfusion strategies^[13,15–19]^ have focused on non-variceal bleeding patients, there has been limited exploration of whether restrictive transfusion has any impact on the safety of patients with esophageal varices after EVL. Considering that low Hb levels may affect the healing of post-ligation ulcers,^[20,21]^ and the availability of blood products is often limited, it is crucial to investigate whether changes in Hb levels affect safety following ligation. Thus, this study aimed to examine the influence of restrictive blood transfusion on the safety of patients with cirrhosis and esophageal varices after EVL, offering insights for improved rebleeding prevention strategies following EVL.

## 2. Methods

### 2.1. Study design

A total of 246 cases were ultimately chosen from among hospitalized patients diagnosed with cirrhosis and esophageal varices who underwent EVL between September 2021 and March 2023 at the Affiliated Hospital of North Sichuan Medical College. In the analysis of early rebleeding following EVL, approximately seventeen variables were considered. The sample size was approximately 10 to 20 times the number of variables. Patients were matched and grouped in a 1:2 ratio based on their post-ligation Hb levels. This study aimed to investigate the impact of restrictive blood transfusion or Hb ≥ 90 g/L on patients with cirrhosis and esophageal varices after EVL, with restrictive blood transfusion defined as maintaining Hb ≥ 60 g/L (according to Chinese guidelines). This study protocol received approval from the Medical Ethical Committee of the Affiliated Hospital of North Sichuan Medical College and adhered to the principles of the Declaration of Helsinki. Written informed consent was waived due to the retrospective nature of this study.

### 2.2. Selection of samples

Inclusion criteria: (a) patients diagnosed with cirrhosis through B-ultrasound or CT and undergoing EVL for emergency bleeding, primary prevention, or secondary prevention of EVB; (b) age 18 years or older; (c) patients with complete basic case data. Exclusion criteria: (a) patients with gastric variceal bleeding; (b) patients with malignant tumors; (c) patients with severe cardiopulmonary and renal diseases; (d) patients with mental disorders; (e) patients with other gastrointestinal bleeding conditions (such as peptic ulcers, tumors, etc).

### 2.3. Data collection

Data were primarily collected through the inpatient electronic medical record system. Information on patients’ demographics, laboratory tests, cirrhosis etiology, Child-Pugh classification, length of hospital stay, transfusion records, endoscopic treatment, and the occurrence of early rebleeding were collected upon admission. Hb levels at admission and the most recent levels before and after EVL were documented. Early rebleeding, complications, and adverse reactions following transfusion were recorded within the 6-week period from hospitalization to discharge.

### 2.4. Primary observation outcome

The primary outcome assessed was early rebleeding, defined as the occurrence of 1 or more of the following symptoms within a period of 72 hours to 6 weeks after endoscopic treatment^[22]^: (a) hematemesis, melena, or hematochezia; (b) a decrease in systolic blood pressure of more than 20 mm Hg or an increase in heart rate of more than 20 beats per minute; (c) a reduction in Hb levels exceeding 30 g/L without the administration of a blood transfusion.^[6]^

### 2.5. Statistical analysis

Statistical analysis was conducted using SPSS® Statistics software version 26.0 for Windows. Continuous variables were presented as either mean ± standard deviation or median and interquartile range, and were compared using Student *t* test or Mann–Whitney *U* tests as appropriate. Categorical variables were expressed as proportions and were assessed using Chi-square or Fisher exact tests. Multivariate analysis was performed to identify risk factors for early rebleeding following EVL. The construction of the nomogram prediction model was accomplished using R software version 4.2. All statistical tests were 2-sided, and a *P* value below 0.05 was considered statistically significant.

## 3. Results

### 3.1. Baseline characteristics and outcomes

**Figure 1.**
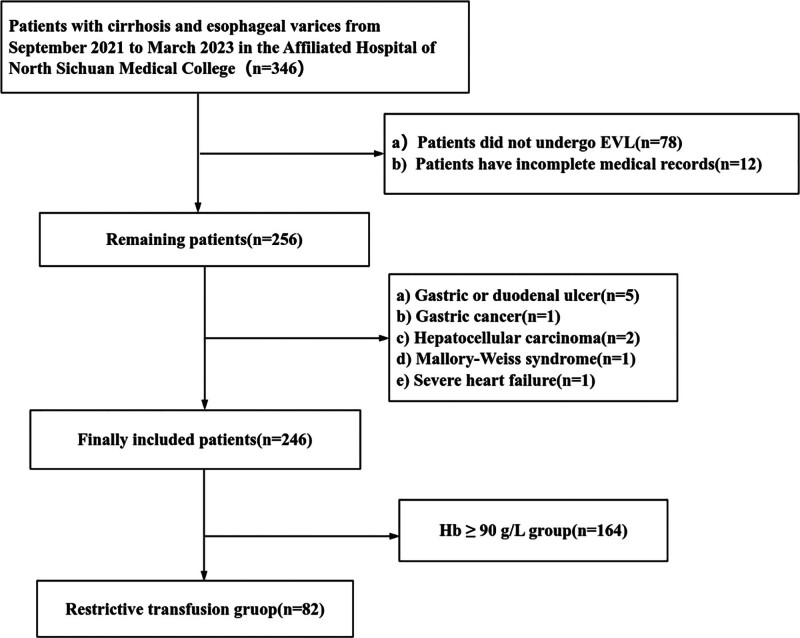
Flowchart illustrating the patient selection process. EVL refer to endoscopic variceal ligation; Hb refer to hemoglobin.

A total of 246 cases were enrolled in this study (Fig. 1). The average age was 56.20 ± 10.95 years, with 157 (63.82%) patients being male. The primary cause of liver cirrhosis in this study was viral hepatitis, particularly hepatitis B virus cirrhosis, accounting for 56.91% (140/246) of cases, followed by alcoholic cirrhosis (6.10%), autoimmune cirrhosis (17.07%), and cryptogenic cirrhosis (19.92%). All patients with hepatitis cirrhosis were diagnosed with chronic hepatitis B, and 131 (93.57%) of them received antiviral therapy. Most patients were classified as Child-Pugh class A or B (93.90%), while hepatic encephalopathy was observed in 5 patients (2.03%). Early post-ligation rebleeding occurred in 10 patients (4.07%). The restrictive RBC transfusion group comprised 82 cases, while the Hb ≥ 90 g/L group included 164 cases. No significant differences were observed between the 2 groups regarding average age, gender distribution, etiology of liver cirrhosis, variceal diameter and severity, portal vein width, and other factors. However, significant disparities were noted between the groups in terms of the presence of red spot signs, the number of patients receiving preventive endoscopic treatment, platelet count, Hb levels, albumin, aspartate aminotransferase, alanine aminotransferase, Child-Pugh class, prothrombin time, international normalized ratio, and the presence of ascites (Table 1).

**Table 1:** General characteristics of patients in both groups.

Patient characteristics	All (n = 246)	Restrictive RBC transfusion (n = 82)	Hb ≥ 90g/L (n = 164)	*P* value
Gender (male/female)	157/89	52/30	105/59	.925
Age (years)	56.20 ± 10.95	57.40 ± 12.34	55.59 ± 10.17	.222
Endoscopic therapy				<.001
Primary prevention	76 (30.89)	5 (6.10)	71 (43.29)	
Secondary prevention	59 (23.98)	4 (4.88)	55 (33.54)	
Emergency hemostasis	111 (45.12)	73 (89.02)	38 (23.17)	
Etiology of cirrhosis				.125
Hepatitis B virus	140 (56.91)	47 (57.32)	93 (56.71)	
Alcoholic	15 (6.10)	8 (9.76)	7 (4.27)	
Autoimmune	42 (17.07)	16 (19.51)	26 (15.85)	
Cryptogenic	49 (19.92)	11 (13.41)	38 (23.17)	
Red color sign (+/−)	205/41	74/8	131/33	.040
Varicose vein diameter (cm)	1.32 ± 0.25	1.35 ± 0.27	1.30 ± 0.24	.144
Degree of varicose vein				.185
Mild/moderate/severe	9/25/212	1/6/75	8/19/137	
Child-Pugh class				<.001
A	147 (59.76)	30 (36.59)	117 (71.34)	
B	84 (34.15)	44 (53.66)	40 (24.39)	
C	15 (6.10)	8 (9.76)	7 (4.27)	
Hb at admission (g/L)	88.00 (69.00,115.00)	65.50 (55.00,75.25)	103.00 (84.00,122.50)	<.001
Pre-ligation Hb (g/L)	86.00 (69.00,113.00)	66.50 (59.00,71.75)	103.00 (81.75,122.50)	<.001
Post-ligation Hb (g/L)	94.00 (80.00,109.75)	71.00 (64.00,79.75)	103.00 (94.00,118.00)	<.001
PLT × 10^9^/L	65.00 (48.00,95.00)	71.00 (49.50,110.75)	62.00 (47.00,89.25)	.028
ALT (U/L)	25.00 (18.00,40.00)	23.00 (15.25,33.75)	26.50 (20.00,40.25)	.043
AST (U/L)	34.00 (26.00,50.75)	29.00 (22.00,50.75)	36.00 (28.75,50.25)	.023
Albumin (g/L)	36.05 ± 6.49	31.40 ± 5.56	38.38 ± 5.63	<.001
Bilirubin (µmol/L)	22.05 (14.55,34.00)	21.25 (14.17,38.08)	22.20 (15.17,33.55)	.916
Creatine (µmol/L)	68.20 (53.92,80.00)	69.00 (53.25,87.92)	67.45 (53.98,79.12)	.439
PT (s)	15.55 (14.20,17.35)	16.70 (15.22,19.28)	14.90 (13.90,16.60)	<.001
INR	1.30 (1.18,1.46)	1.40 (1.27,1.61)	1.24 (1.15,1.40)	<.001
Width of portal vein (cm)	1.40 (1.20,1.50)	1.35 (1.10,1.50)	1.40 (1.20,1.50)	.776
Ascites (+/−)	115/131	49/33	66/98	.004

ALT = alanine aminotransferase, AST = aspartate aminotransferase, Hb = hemoglobin, INR = international normalized ratio, PLT = platelet, PT = prothrombin time, RBC = red blood cell.

There were 10 patients who experienced early rebleeding, with 7 patients (8.54%) in the restrictive transfusion group and 3 patients (1.83%) in the Hb ≥ 90 g/L group. A significant difference in the incidence of early rebleeding was observed between the 2 groups. The incidence of hepatic encephalopathy was similar in both groups. Additionally, the length of hospital stay and hospitalization costs were higher in the restrictive transfusion group compared to Hb ≥ 90 g/L group (Table 2).

**Table 2:** Comparison of the main outcomes between both groups.

Outcomes	All (n = 246)	Restrictive RBC transfusion (n = 82)	Hb ≥ 90 g/L (n = 164)	*P* value
Early rebleeding	10 (4.07)	7 (8.54)	3 (1.83)	.030
Hepatic encephalopathy	5 (2.03)	3 (3.66)	2 (1.22)	.424
Adverse reactions to transfusion	7 (2.85)	5 (6.10)	2 (1.22)	.078
Mean length of admission (days)	11.00 (10.00, 14.00)	13.00 (10.00, 17.00)	11.00 (9.00, 13.00)	<.001
Hospitalization expenses	14180.12 (10753.40, 20457.52)	20752.14 (16320.18, 28463.57)	12047.19 (9836.10, 15359.35)	<.001

RBC = red blood cell.

### 3.2. Different post-ligation Hb levels and early rebleeding

To investigate the relationship between post-ligation Hb levels and the rate of early rebleeding, post-ligation Hb was categorized into 3 subgroups: 60 g/L ≤ Hb < 70 g/L, 70 g/L ≤ Hb < 90 g/L, and Hb ≥ 90 g/L. The results revealed significant differences in the occurrence of early rebleeding. Patients with lower post-ligation Hb levels were more likely to experience early rebleeding (8.57% vs 8.51% vs 1.83%, *P* = .024) (Table 3).

**Table 3:** Comparison of early rebleeding rates at different hemoglobin levels.

Variables	All (n = 246)	60 g/L ≤ Hb < 70 g/L (n = 35)	Hb ≥ 70 g/L (n = 47)	Hb ≥ 90 g/L (n = 164)	*P* value
Early rebleeding	10 (4.07)	3 (8.57)	4 (8.51)	3 (1.83)	.024

Hb = hemoglobin.

### 3.3. Univariate and multivariate analysis of early rebleeding after EVL

As demonstrated in Tables 4 and 5, univariate analysis indicated that age, gender, varicose vein diameter, etiology of liver cirrhosis, and the degree of ascites did not exhibit statistical significance. However, patients who experienced early rebleeding were more likely to have lower post-ligation Hb levels (73.00 g/L vs 95.00 g/L, *P* = .004), lower pre-ligation albumin levels (30.52 g/L vs 36.29 g/L, *P* = .006), a higher Child-Pugh class (class C 30% vs 5.08%, *P* = .025), and an elevated baseline international normalized ratio (1.51 vs 1.29, *P* = .022). In general, early rebleeding patients received more RBC and plasma transfusions than those without rebleeding. Eliminating the multiple factors, the number of RBC transfusions, Child-Pugh class, Hb levels, albumin levels, and the volume of plasma and RBC transfusions after EVL were selected for further logistic regression analysis. The results (using backward Wald) indicated that Hb (OR = 0.96, 95% CI: 0.95–0.97; *P* < .001) and Child-Pugh class C (OR = 6.37, 95% CI: 1.28–31.67; *P* = .024) were independent risk factors for early rebleeding after EVL. This suggests that higher Hb levels are associated with a reduced risk of rebleeding after EVL, and patients with Child-Pugh class C have a higher risk of rebleeding compared to those with class A. Forward Wald regression analysis revealed that albumin (OR = 0.90, 95% CI: 0.88–0.93; *P* < .001) was a contributing factor to early rebleeding. When analyzing Hb levels between 60 g/L and 90 g/L (restrictive blood transfusion) or Hb ≥ 90 g/L, the results suggested that restrictive RBC transfusion (OR = 4.61, 95% CI: 1.06–19.99; *P* = .041) appeared to increase the risk of early rebleeding after EVL.

**Table 4:** Comparison of general data between patients with and without early rebleeding after EVL.

Patient characteristics	Rebleeding (n = 10)	No rebleeding (n = 236)	*P* value
Age (years)	57.10 ± 11.10	56.16 ± 10.97	.790
Gender (male/female)	8/2	149/87	.453
Varicose vein diameter (cm)	1.43 ± 0.27	1.31 ± 0.25	.166
Endoscopic therapy			.344
Primary prevention	2 (20.00)	74 (31.36)	
Secondary prevention	1 (10.00)	58 (24.58)	
Emergency hemostasis	7 (70.00)	104 (44.07)	
Etiology of cirrhosis			.681
Hepatitis B virus	6 (60.00)	134 (56.78)	
Alcoholic	1 (10.00)	14 (5.93)	
Autoimmune	2 (20.00)	40 (16.95)	
Cryptogenic	1 (10.00)	48 (20.34)	
Pre-ligation Hb (g/L)	69.00 (67.00, 75.50)	87.50 (69.00, 115.00)	.086
Post-ligation Hb (g/L)	73.00 (67.25, 86.50)	95.00 (81.00, 111.00)	.004
Bilirubin (µmol/L)	27.55 (20.20, 50.20)	21.09 (14.40, 34.00)	.095
Creatine (µmol/L)	68.30 (57.38, 115.15)	68.20 (53.55, 80.00)	.417
PT (s)	17.40 (15.27, 19.23)	15.50 (14.17, 17.20)	.061
INR	1.51 (1.34, 1.69)	1.29 (1.18, 1.45)	.022
Albumin (g/L)	30.52 ± 6.26	36.29 ± 6.41	.006
Child-Pugh class			.025
A	4 (40.00)	143 (60.59)	
B	3 (30.00)	81 (34.32)	
C	3 (30.00)	12 (5.08)	
Number of RBC transfusion	7 (70.00)	75 (31.78)	.030
Number of plasma transfusion	7 (70.00)	85 (36.02)	.066
Transfusion pre-ligation			
RBC	0.00 (0.00, 0.00)	0.00 (0.00, 0.00)	.338
Plasma	0.00 (0.00, 0.00)	0.00 (0.00, 0.00)	.525
Transfusion post-ligation			
RBC	3.25 (0.50, 4.38)	0.00 (0.00, 0.00)	<.001
Plasma	300.00 (50.00, 1112.50)	0.00 (0.00, 300.00)	.012

EVL = endoscopic variceal ligation, Hb = hemoglobin, INR = international normalized ratio, PT = prothrombin time, RBC = red blood cell.

**Table 5:** Multivariate analysis of risk factors for early rebleeding after EVL.

Variables	B	S.E.	Z	*P*	OR (95% CI)
Hemoglobin	−0.039	0.006	42.792	<.001	0.961 (0.950–0.973)
Child-Pugh C	1.852	0.818	5.125	.024	6.372 (1.282–31.667)

### 3.4. Construction of the nomogram prediction model

**Figure 2.**
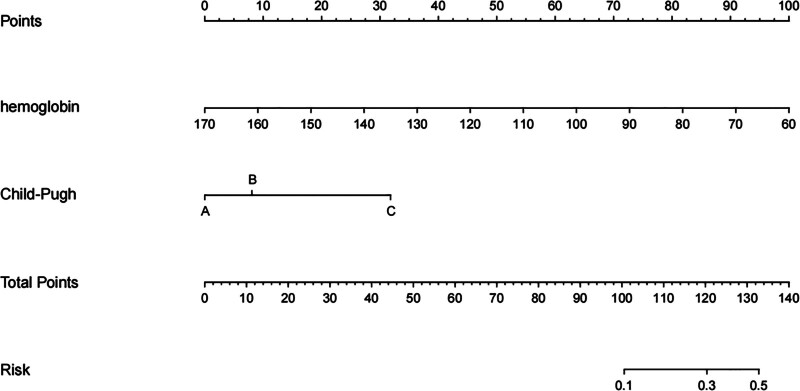
Nomogram prediction model for early rebleeding after EVL. EVL refer to endoscopic variceal ligation.

A nomogram prediction model was developed based on the results obtained from logistic multivariate analysis. The values of the 2 independent risk factors were mapped upwards to the integral line located at the top of the nomogram, yielding the corresponding integrals. Subsequently, the integrals for all independent influencing factors were aggregated to calculate the total score. By referring to the predictive line at the bottom of the nomogram (Fig. 2), the predictive probability of early rebleeding after EVL in patients with cirrhotic esophageal varices could be determined.

## 4. Discussion

Cirrhosis complicated with acute EVB is a common critical condition that significantly contributes to high morbidity and mortality among cirrhotic patients. This study was conducted to investigate whether restrictive transfusion has any impact on the safety of patients with esophageal varices after EVL. The analysis revealed that restrictive RBC transfusion, Hb, albumin, and Child-Pugh class C were influential factors of early rebleeding. It was concluded that restrictive blood transfusion appeared to increase the risk of early rebleeding in cirrhotic patients after EVL.

In the current study, we observed 10 cases of early rebleeding after EVL, which was a lower incidence compared to findings in previous research. Specifically, there were 7 patients (8.54%) in the RBC transfusion group and 3 patients (1.83%) in the Hb ≥ 90 g/L group, a pattern consistent with prior reports.^[18]^ It is important to note that the RBC transfusion group had a higher proportion of patients with Child-Pugh class B, ascites, lower Hb levels, and a greater utilization of plasma and albumin transfusions, indicating that the general condition of these patients was more severe compared to those in the Hb ≥ 90 g/L group. Additionally, the restrictive transfusion group had a relatively larger proportion of patients with acute hemorrhage, while the Hb ≥ 90 g/L group predominantly received preventive treatment. This difference in the approach to treatment aligns with previous literature^[19]^ and contributes to the variation in early rebleeding rates between the 2 groups. EVB can lead to a decrease in Hb levels and the loss of coagulation factors. In patients with cirrhosis, there is compromised liver function and reduced synthesis of coagulation factors, further increasing the risk of rebleeding. The study results also highlighted significant disparities between the restrictive transfusion group and the Hb ≥ 90 g/L group in terms of the length of hospital stay and hospitalization costs. This difference may be attributed to the increased costs associated with blood transfusions and prolonged hospital stays due to the severity of illness in the restrictive transfusion group. While the univariate analysis suggested that the type and quantity of blood transfusions were potential risk factors for early rebleeding after EVL, these associations were not observed in the results of the multivariate analysis. Further research is needed to explore these relationships in more detail.

Previous studies have consistently demonstrated that Hb levels play a crucial role in predicting early rebleeding after EVL.^[20,21,23]^ In the context of blood transfusion’s impact on post-EVL safety, this study also delved into the influence of RBC transfusion aimed at maintaining different post-ligation Hb levels (60 g/L ≤ Hb < 70 g/L, 70 g/L ≤ Hb < 90 g/L, and Hb ≥ 90 g/L) on the occurrence of early rebleeding following EVL. The early rebleeding rates in these 3 groups were 8.57%, 8.51%, and 1.83%, respectively, highlighting that cirrhotic patients with lower Hb levels are more prone to rebleeding. However, there was no significant difference observed between the groups with Hb levels ranging from 60 g/L to 70 g/L and 70 g/L to 90 g/L. It is worth noting that the high Hb level in patients after ligation might promote the healing of post-ligation ulcers. Nonetheless, it cannot be ruled out that other factors such as liver function classification, the presence of ascites, and low albumin levels could also contribute to these results. Further prospective studies are warranted to explore this aspect in more detail. However, pairwise comparison analysis did not provide conclusive evidence of differences in rebleeding rates between these groups. While this study established a connection between Hb levels and post-ligation rebleeding rates, it did not determine the most appropriate range of Hb levels, which could be a valuable subject for exploration in prospective randomized controlled trials.

This study revealed significant disparities between the 2 groups in terms of post-ligation Hb, international normalized ratio, serum albumin, Child-Pugh class, RBC transfusion status, and the volume of plasma and RBC transfused after ligation. Prior research has established a close relationship between the severity of liver cirrhosis and prognosis.^[24–27]^ Higher Child-Pugh grades are associated with an increased risk of early rebleeding, particularly in cases of Child-Pugh class C, a finding that was also confirmed by this study. Considering the numerous interrelated factors at play, the number of RBC transfusions, Child-Pugh class, Hb levels, albumin levels, and the volume of plasma and RBC transfusions after EVL were selected for further logistic regression analysis. The results of this analysis indicated that Hb levels and Child-Pugh class C were independent factors influencing the risk of rebleeding after EVL.

Current guidelines recommend albumin as the preferred volume expander in cirrhotic patients with ascites.^[5,28]^ Albumin has been proven to effectively improve hypovolemia, prevent circulatory dysfunction following paracentesis, and enhance outcomes in patients with conditions like spontaneous bacterial peritonitis or hepatorenal syndrome.^[29–32]^ However, the role of albumin in managing EVB in cirrhotic patients remains less clear. A multicenter retrospective study conducted by Zhou et al^[33]^ found that maintaining an albumin concentration above 31.5 g/L was a protective factor against early rebleeding following EVL. Another retrospective study by Wang et al^[34]^ demonstrated that in patients with hypoalbuminemia, albumin infusion could reduce the risk of in-hospital rebleeding. Furthermore, in patients with Child-Pugh class C cirrhosis, albumin infusion was associated with a decreased number of in-hospital deaths. The univariate analysis in our study also indicated that rebleeding after EVL may be linked to low albumin levels, a finding that was further supported by multivariate regression analysis (utilizing Forward Wald). Considering that pre-ligation blood transfusion may increase the risk of rebleeding, it is important to recognize the potential benefits of correcting low albumin levels within 3 days after EVL, before the formation of ligation ulcers. This correction can promote ulcer healing and improve the hemostatic effect, potentially contributing to better patient outcomes.

The results of the univariate analysis in this study suggested that restrictive blood transfusion appeared to be associated with an increased risk of early rebleeding after EVL in cirrhotic patients. When we specifically analyzed the impact of maintaining Hb levels between 60 g/L and 90 g/L (indicative of restrictive blood transfusion) or having Hb ≥ 90 g/L, restrictive blood transfusion continued to be a significant factor influencing rebleeding after EVL. Furthermore, when Child-Pugh class, Hb, albumin, and other factors were included in the multivariate regression analysis, the results highlighted that Hb was also a key factor influencing the risk of early rebleeding after EVL. These findings collectively indicate that restrictive RBC transfusion might elevate the risk of early rebleeding. Notably, the study revealed that higher Hb levels, especially in patients with Hb levels exceeding 90 g/L, were associated with a reduced risk of early rebleeding. This observation further supports the idea that restrictive transfusion strategies might increase the risk of rebleeding after ligation. Additionally, the study found that patients with Child-Pugh class C cirrhosis had a significantly higher risk of early rebleeding after EVL when compared to patients with Child-Pugh class A. To further assess the relationship between Hb levels, Child-Pugh class, and the occurrence of early rebleeding after EVL in cirrhotic patients with esophageal varices, a nomogram prediction model was constructed. This model assigns a corresponding score to each independent influencing factor, calculates the risk prediction value of early rebleeding based on the total score, and transforms the complex regression equation into a visual graph. This visual representation of the data is more intuitive and practical, facilitating a better understanding of the relationships among these factors. If the predicted probability of early rebleeding is high, improving liver function, albumin infusion, and RBC transfusion are of great significance to prevent early rebleeding after EVL. However, we did not validate the model, which is the deficiency of this study and needs to be improved in future research.

This study has several limitations that should be acknowledged. Firstly, it was conducted as a single-center clinical review, which resulted in a relatively small sample size and potential bias. Ideally, there should have been 3 groups: no transfusion, restrictive transfusion, and liberal transfusion groups. However, due to consensus and ethical principles, the control group that was supposed to receive RBC transfusion to maintain Hb ≥ 90 g/L did not receive blood transfusion. The higher rebleeding rate in the restrictive blood transfusion group may have been influenced by poorer liver function, such as lower albumin levels, compared to the Hb ≥ 90 g/L group, introducing significant bias. Therefore, to reduce rebleeding events and conserve blood products, a greater emphasis should be placed on performing prophylactic esophageal variceal ligation, and randomized controlled trials are warranted to further investigate the impact of transfusion strategies on cirrhotic patients after ligation. Secondly, this study did not record whether patients had a history of variceal bleeding in the past or their endoscopic manifestations during ligation, such as the presence of high-risk lesions, which could potentially influence post-ligation rebleeding. Finally, the study did not analyze the mortality rate, as the status of some patients after automatic discharge was unclear.

In conclusion, restrictive blood transfusion (maintaining Hb at 60–90 g/L) appeared to increase the risk of early rebleeding in cirrhotic patients after EVL. However, the lack of a true control group that receive the recommended transfusion strategy introduces potential bias, and further research is needed to confirm these findings in future studies.

## Author contributions

**Data curation:** Yufeng Chen, Wen Ming, Jingjing Chen, Xi Wang.

**Formal analysis:** Yufeng Chen, Wen Ming, Jingjing Chen, Xi Wang, Guobin He.

**Investigation:** Yufeng Chen.

**Methodology:** Yufeng Chen.

**Supervision:** Guobin He.

**Validation:** Guobin He.

**Writing – original draft:** Yufeng Chen, Wen Ming, Guobin He.

**Writing – review & editing:** Yufeng Chen, Guobin He.
